# A study on the quality of life and burden of informal caregivers of children with metachromatic leukodystrophy in Poland

**DOI:** 10.1186/s13023-026-04545-6

**Published:** 2026-08-11

**Authors:** Dariusz Walkowiak, Tomasz Grybek, Karolina Śledzińska, Anna Lemska, Ninela Irga-Jaworska, Jolanta Wierzba, Maria Mazurkiewicz-Bełdzińska, Jan Domaradzki

**Affiliations:** 1 Department of Organization and Management in Health Care, Poznan University of Medical Sciences, Poznan, Poland; 2 Department of Public Health and Social Medicine, Medical University of Gdansk, Gdansk, Poland; 3 Borys the Hero Foundation, Gdansk, Poland; 4 Department of Paediatrics, Haemathology and Oncology, Medical University of Gdansk, Gdansk, Poland; 5 Division of Developmental Neurology, Medical University of Gdansk, Gdansk, Poland; 6 Division of Internal and Pediatric Nursing, Medical University of Gdansk, Gdansk, Poland; 7 Department of Social Sciences and Humanities, Poznan University of Medical Sciences, Rokietnicka 7, Poznan, 60-806 Poland

**Keywords:** Caregiver burden, Family caregivers, Metachromatic leukodystrophy, Parents' experiences, Quality of life

## Abstract

**Background:**

Metachromatic leukodystrophy (MLD) is a rare, inherited lysosomal storage disorder caused by a deficiency in arylsulfatase A (ARSA), leading to sulfatide accumulation, demyelination, and progressive neurological decline. Without treatment, patients – particularly those with early-onset forms – typically lose the ability to walk or communicate within months of symptom onset, and life expectancy is markedly reduced, often below 10 years. Although MLD is likely underdiagnosed in Poland, prevalence may be higher than average. Early diagnosis and prompt intervention with gene therapy (arsa-cel) are critical, as neuronal damage is irreversible. This exploratory study aimed to characterise the clinical course of MLD in Poland and assess the quality of life and financial well-being of caregivers.

**Results:**

The study included 13 MLD patients and 28 caregivers. The most common forms were late-infantile (46.2%) and early-juvenile (38.5%). Most patients were classified as GMFC-MLD stage 6 (53.8%) and ELFC-MLD stage 4 (69.2%). Common symptoms included spasticity (84.6%), walking difficulties (84.6%), and speech disorders (69.2%). The mean age at diagnosis was 4.8 years, with 69.2% of caregivers reporting that delayed diagnosis adversely impacted the child’s health. Caregivers were predominantly female (67.9%) and reported high caregiving demands (mean = 35.7 h/week). WHOQOL-BREF scores exceeded medians for caregivers of people with rare diseases across all domains. Financial well-being was strongly correlated with physical health (*r* = 0.636, *p* < 0.001) and psychological health (*r* = 0.717, *p* < 0.001). Only 14.3% of MLD caregivers received psychological counselling at diagnosis, though 92.3% received financial support. Gene therapy was reported in 15.4% of cases; most patients received multiple forms of rehabilitative therapy.

**Conclusion:**

MLD in Poland is characterised by early-onset, rapidly progressing forms that lead to severe disability and impose significant burdens on families. Delayed diagnosis was commonly reported in this cohort, with many caregivers indicating that it had negatively affected the child’s health and limited timely access to potentially disease-modifying treatments. Despite intensive caregiving responsibilities, many caregivers report a relatively preserved quality of life, especially when financial support is available. While these exploratory findings should be interpreted with caution, they support the need for improved early detection and coordinated care for individuals with MLD. Within this broader context, systematic newborn screening in Poland may facilitate earlier diagnosis and timely access to gene therapy, potentially improving patient outcomes and reducing the long-term burden on families and the healthcare system.

**Clinical trial number:**

Not applicable

## Background

Metachromatic leukodystrophy (MLD; ICD-10: E75.2; ICD-11: 5C56.02; OMIM: #156310, #249900, #250100, ORPHA: 512) is a rare, neurodegenerative lysosomal storage disorder caused by a deficiency of the enzyme arylsulfatase A (ARSA) [1–3]. It is inherited in an autosomal recessive manner, in which the presence of two biallelic pathogenic variants in the *ARSA* gene is the primary cause of the deficient enzyme activity and subsequent accumulation of sulfatides within cells of the central and peripheral nervous systems, resulting in progressive demyelination [4, 5]. In rare cases, MLD may also result from mutations in the *PSAP* gene, which encodes prosaposin – the precursor of saposin B, an essential activator of ARSA – leading to impaired enzyme function despite normal ARSA levels [6, 7]. Pathological sulfatide accumulation leads to severe motor and cognitive decline and ultimately to premature death, with the most rapid progression observed in early-onset forms [8].

The incidence of MLD varies significantly across different populations, ranging from approximately 1 in 40,000-160,000. Studies indicate that the lowest reported birth prevalence, considering both prenatal and postnatal diagnosis, is found in the Czech Republic at 0.69 per 100,000 births, whereas the highest is reported in Poland at 4.1 per 100,000 births [2, 9]. When only postnatal cases are considered, Sweden shows the highest incidence at 2.5 per 100,000 births, while Japan has the lowest incidence at 0.16 per 100,000 births [2, 10]. In a 2011 study, Ługowska et al. [9] investigated the frequency of common *ARSA* gene mutations – specifically c.459 + 1G > A, p.P426L, and p.I179S – in the Polish population. They identified carrier rates suggesting an expected MLD birth prevalence of about 1 in 25,000. This estimated prevalence was notably higher than the observed rate of diagnosed cases, indicating that MLD might be underdiagnosed in Poland. The study also noted that the p.I179S mutation was more prevalent among controls than patients, suggesting that individuals with this mutation might present with milder or atypical symptoms, potentially leading to underdiagnosis [9, 11].

MLD is classified into four main clinical forms according to the age of symptom onset: late infantile (LI), early juvenile (EJ), late juvenile (LJ), and adult-onset (AO) [3, 8]. The LI form is the most prevalent and severe, accounting for 50–60% of all MLD cases, with onset typically before 30 months of age, usually between 15 and 24 months. EJ onset occurs between 30 months and 7 years, LJ onset between 7 and 17 years, and AO after 17 years. Symptoms of the LI form often include delays in gross and fine motor skills, loss of speech, muscle hypotonia, ataxia, and spasticity, eventually resulting in the complete loss of motor and cognitive abilities. EJ presents with more variability, but once the patient loses independent walking ability, the progression of motor and cognitive decline resembles that of the infantile form [11–13].

Early symptom onset is associated with faster and more severe disease progression in MLD. The median time from symptom onset to the loss of independent walking (beyond GMFC-MLD stage one) is approximately 2.7 months for LI patients and seven months for EJ patients [14–18]. Without treatment, median survival for untreated LI patients is 8.42 years, while 68.6% of EJ patients survive 15 years after symptom onset [2, 13].

Treatment options for MLD were limited before the approval of atidarsagene autotemcel (arsa-cel), an autologous CD34 + hematopoietic stem/progenitor cells (HSPC) gene therapy approved in the EU in December 2020 and in the UK in January 2021 [19, 20]. Arsa-cel involves collecting HSPCs from mobilised peripheral blood, genetically modifying them with a lentiviral vector (ARSA LVV), and reintroducing them to produce functional ARSA enzyme. The gene modification integrates into the cell’s genome, ensuring the corrected gene is passed to progeny cells. Arsa-cel is approved for patients with *ARSA* gene mutations, specifically pre-symptomatic LI and EJ, and early symptomatic EJ patients who can still walk independently [21, 22].

Early diagnosis and treatment are critical in MLD due to its rapid and irreversible progression. Without newborn screening (NBS), many patients are diagnosed too late to benefit from gene therapy [23]. Recent advances in NBS enable early detection with high specificity [24–27]. Newborn screening for MLD has also been shown to be a cost-effective approach to improving patient outcomes [28, 29].

Most untreated early-onset MLD patients live much of their remaining lives with severe disabilities, leading to significant caregiving burden and high healthcare resource utilisation within the healthcare system [13, 30]. The burden on caregivers of MLD patients is substantial, affecting their physical health, mental well-being, and social lives. Many caregivers experience anxiety, depression, and reduced quality of life (QoL) compared to the general population [27, 31–33]. Professionally, 76.5% of caregivers reported stopping work or reducing their working hours, often leading to adverse career impacts [32]. The findings underscore the need for better support systems for MLD-affected families and suggest broader applicability to other rare lysosomal storage disorders.

While access to molecular diagnostics, including NBS, and advanced therapies such as gene therapy, is essential in the context of MLD, it is not sufficient, particularly because MLD affects not only the patient but also profoundly impacts the entire family. Since caregivers face a multidimensional burden: psychological, physical, social, and financial, this study aimed to analyse the experiences of Polish families affected by MLD, focusing on the types of MLD present in the population, the most common symptoms, disease severity, and the caregiving burden. By examining caregivers’ QoL and perceived financial well-being, the study highlights the urgent need for a shift in healthcare and social policy: one that systematically includes caregivers within the scope of support.

## Methods

### Study design

The study was designed as a computer-assisted, self-administered, anonymous online survey to assess the QoL and financial well-being of informal caregivers of persons with MLD [34]. Because, to date, there was no registry of patients with MLD in Poland, the study was designed as patient-oriented [35], i.e. it was developed and conducted in close collaboration with the patient advocacy organisation the Borys the Hero Foundation, which aims at spreading the knowledge and public awareness on MLD, and develops social activism in the field of rare diseases, and leukodystrophy in particular. Thus, both the study design, the development of the questionnaire, and data collection involved family caregivers as full-fledged research collaborators.

The study’s design was approved by the Poznan University of Medical Sciences Bioethics Committee (KB – 145/25, from 21.02.2025). The study was also approved by the Borys the Hero Foundation, and in compliance with the Declaration of Helsinki (as amended in 2000) [36], before completing the survey, every participant provided written informed consent.

### Participants

Due to the rare nature of the disease and the lack of a patient registry for MLD in Poland, participant recruitment was conducted with the assistance of the patient advocacy organisation, the Borys the Hero Foundation. The foundation recruited caregivers through internal communication channels, including its internal mailing and WhatsApp group for MLD caregivers.

The eligibility criteria for participation in the study included: (1) being at least 18 years old, (2) being a parent or other family caregiver of a person with MLD, (3) giving direct care to a person with a MLD diagnosis confirmed by a molecular testing, (4) speaking Polish, (5) ability to use electronic devices necessary for participation in the online study, and (6) providing written informed consent to participate in the study.

### Questionnaire

The questionnaire used in this survey comprised four sections. The first part included questions on the clinical characteristics of a person with MLD, including diagnosis, symptoms, diagnostic process, and therapies. Caregivers also assessed motor and communication function using the Gross Motor Function Classification for MLD (GMFC-MLD), which categorizes motor impairment from mild difficulties (level 1) to complete loss of mobility (level 6) [14, 18], and the Expressive Language Function Classification for MLD (ELFC-MLD), which measures the regression of expressive language abilities from E0 (normal communication) to E4 (complete loss of expressive language) [17].

The second part of the questionnaire asked about caregivers’ assessment of their perceived QoL. The abbreviated Polish version of the World Health Organisation Quality of Life-BREF (WHOQOL-BREF) was used to assess it [37]. This tool, commonly used in clinical trials and epidemiological studies, consists of 24 items across four domains: physical health (7 items, raw score range: 7–35), psychological health (6 items, raw score range: 6–30), social relationships (3 items, raw score range: 3–15), and environmental (8 items, raw score range: 8–40). It also includes two questions on the overall QoL and health satisfaction. Responses are given on a 5-point Likert scale, with higher scores indicating better QoL.

The third part included the Consumer Financial Protection Bureau Financial Well-Being Scale (CFPB-FWBS) [38, 39], a ten-question tool assessing financial security and freedom of choice. As no Polish version existed, it was back-translated and adapted. Pre-testing with caregivers of chronically ill children and healthy adults confirmed its sensitivity and reliability. The final part of the questionnaire included single-choice, closed-ended questions about the demographic data of the parent/caregiver of a person with MLD.

### Data collection

The study was conducted over four weeks, from February to March 2025, through WhatsApp, an instant messenger, social media, and the internal mailing of the Borys the Hero Foundation. In the first stage, all caregivers received an invitation letter to take part in the study, which included information about the research team, the study’s objectives, its methodology, and how the results would be presented. They were also informed about the anonymous, voluntary, and confidential nature of the study. After signing the electronic informed consent form, all eligible participants gained access to the online questionnaire.

No financial compensation was provided for participation in the study. Completing the survey required approximately 15–20 min.

### Data analysis

Descriptive statistics were used to present the data. The normality of the distribution of variables was assessed using the Shapiro-Wilk test. The Brown-Forsythe test was used to check the assumption of equal variances between the compared populations. Group comparisons were conducted using Welch’s t-test, with a significance threshold set at *p* < 0.05. The relationships between WHOQOL domains and CFPB-FWBS were examined using Pearson’s correlation. WHOQOL domain cut-off points were based on national medians, and participants were categorised into two groups. Statistical analyses were performed using JASP (Version 0.19.3).

## Results

Table 1 presents the demographic and clinical characteristics of the study participants. The cohort included 13 patients, with a slight predominance of females (53.8%) over males (46.2%). Participants ranged in age from 4 to 22 years, with a mean of 11.9 years (SD = 5.5) and a median of 13 years. In terms of symptom onset, 46.2% of patients had LI onset, 38.5% had EJ onset, while LJ and AO were each observed in 7.7% of cases.

Functional status was assessed using the GMFC-MLD, with 53.8% of patients classified as stage 6, 23.1% as stage 5, 7.7% as stage 4, and 15.4% as stage 0. The ELFC-MLD showed that most patients (69.2%) were at stage 4, while stages 0, 1, and 3 were represented by 15.4%, 7.7%, and 7.7% of patients, respectively.

**Table 1:** Patients’ demographics

Characteristic	*N*(%)
*Gender*	
Female	7 (53.8)
Male	6 (46.2)
*Age (in years)*	
Range	4–22
Mean	11.9
SD	5.5
Median	13
*Time of symptom onset*	
Late infantile (LI)	6 (46.2)
Early juvenile (EJ)	5 (38.5)
Late juvenile (LJ)	1 (7.7)
Adult-onset (AO)	1 (7.7)
*GMFC-MLD*	
0	2 (15.4)
1	0
2	0
3	0
4	1 (7.7)
5	3 (23.1)
6	7 (53.8)
*ELFC-MLD*	
0	2 (15.4)
1	1 (7.7)
2	0
3	1 (7.7)
4	9 (69.2)
*Treatment used for the person with MLD?*	
Bone marrow/stem cell transplant	6 (46.2)
Enzyme replacement therapy	1 (7.7)
Gene therapy	2 (15.4)
None	4 (30.8)

The age at MLD diagnosis ranged from 2 to 14 years (mean = 4.8, SD = 3.5; median = 4) (Table 2). Before confirmation, 10 patients had no prior diagnoses, one had one, and two had 2–3. Delayed diagnosis was reported to negatively impact health by 69.2%, while 15.4% were unsure, and 7.7% said “rather no” or “definitely no.”

The most common symptoms were spasticity (84.6%), walking difficulties (84.6%), motor coordination loss (69.2%), and speech disorders (69.2%). Other symptoms included dysautonomia and cognitive decline (61.5% each), irritability and gastrointestinal issues (53.8%), pain and concentration problems (46.2%), dystonia and frequent infections (30.8%), and emotional instability (7.7%). One respondent (7.7%) reported no symptoms.

Therapies included physical rehabilitation (92.3%), speech therapy (61.5%), occupational or sensory integration therapy (53.8%), Augmentative and Alternative Communication (AAC) (46.2%), seizure treatment (38.5%), pain management (23.1%), feeding therapy and osteopathy (15.4% each), and hydrotherapy (30.8%). One respondent (7.7%) reported no therapy. Rehabilitation time ranged from 0 to 29 h/week (mean = 9.4, SD = 8; median = 7). Clinical trial participation was reported by 38.5%, and 61.5% were under expert or multidisciplinary care.

**Table 2:** Characteristics of patients in their mothers’ opinions

Characteristic	*N* (%)
*Age at MLD diagnosis (in years)*	
Range	2–14
Mean	4.8
SD	3.5
Median	4
*Number of previous diagnoses before MLD confirmation?*	
0	10
1	1
2–3	2
*Delayed diagnosis had a negative impact on the patient’s health*	
Definitely yes	9 (69.2)
Rather yes	0
I don’t know / no opinion	2 (15.4)
Rather no	1 (7.7)
Definitely no	1 (7.7)
MLD symptoms in the patient*	
Epilepsy	5 (38.5)
Spasticity	11 (84.6)
Loss of motor coordination	9 (69.2)
Walking difficulties	11 (84.6)
Dysautonomia (excessive salivation)	8 (61.5)
Dystonia	4 (30.8)
Pain	6 (46.2)
Frequent infections	4 (30.8)
Gastrointestinal problems	7 (53.8)
Visual impairment	3 (23.1)
Hearing loss	0
Irritability	7 (53.8)
Emotional instability	1 (7.7)
Intellectual decline	8 (61.5)
Memory loss	3 (23.1)
Difficulty concentrating	6 (46.2)
Speech disorders	9 (69.2)
None	1 (7.7)
Types of therapy used*	
Pain management	3 (23.1)
Antiepileptic treatment	5 (38.5)
Physical rehabilitation / physiotherapy	12 (92.3)
Speech therapy / speech training / neurological speech therapy	8 (61.5)
Occupational therapy	7 (53.8)
Psychological therapy	4 (30.8)
Augmentative and alternative communication	6 (46.2)
Feeding therapy	2 (15.4)
Manual therapy	5 (38.5)
Sensory integration therapy	7 (53.8)
Osteopathy	2 (15.4)
Hydrotherapy, pool/water therapy	4 (30.8)
None	1 (7.7)
*Hours per week dedicated to rehabilitation*	
Range	0–29
Mean	9.4
SD	8
Median	7
*The patient participated in clinical trials*	
Yes	5 (38.5)
No	8 (61.5)
*Under the care of an expert/multidisciplinary centre*	
Yes	8 (61.5)
No	5 (38.5)

* It was possible to give more than one answer

Table 3 presents demographic and socioeconomic data of MLD caregivers. Most were female (67.9%), with males comprising 32.1%. The largest age group was 40–49 years (46.4%), followed by 20–29 and 30–39 years (each 14.3%). Smaller groups were aged 50–59, 60–69, and 70+ (each 7.1%), and one caregiver (3.6%) was 16–19 years old. Caregivers were mostly mothers (46.4%), fathers (28.6%), grandmothers (14.3%), spouses (7.1%), and other relatives (3.6%).

Professionally, 42.9% worked full-time, 10.7% part-time, 25% were unemployed due to caregiving, 14.3% were retired/pensioners, 3.6% were unemployed for other reasons, and 3.6% were in school. Financial well-being (CFPB scale) was rated very high/high (21.4%), medium-high (21.4%), medium-low (21.4%), low (10.7%), and very low (3.6%).

Care time ranged from 0 to 110 h/week (mean = 35.7, SD = 35.3; median = 24.5). Only 14.3% received psychological counselling at diagnosis, while 85.7% did not. Financial support was received by 92.3% and institutional support by 30.8%.

**Table 3:** Caregivers’ demographics

Characteristic	*N*/*N*(%)
*Gender*	
Female	19 (67.9)
Male	9 (32.1)
*Age*	
16–19	1 (3.6)
20–29	4 (14.3)
30–39	4 (14.3)
40–49	13 (46.4)
50–59	2 (7.1)
60–69	2 (7.1)
70+	2 (7.1)
*Relationship with an MLD patient*	
Mother	13 (46.4)
Father	8 (28.6)
Grandmother	4 (14.3)
Spouse	2 (7.1)
Other family member	1 (3.6)
*Professional activity*	
Full-time employment	12(42.9)
Part-time employment	3 (10.7)
Unemployed due to caregiving responsibilities	7 (25)
Unemployed	1 (3.6)
Retired/pensioner	4 (14.3)
Student (still in school)	1 (3.6)
*CFPB Financial Well-Being*	
Very low	1 (3.6)
Low	3 (10.7)
Medium low	6 (21.4)
Medium high	6 (21.4)
High	6 (21.4)
Very high	6 (21.4)
*Hours per week spent caring for an MLD patient*	
Range	0-110
Mean	35.7
SD	35.3
Median	24.5
*Psychological counselling received during diagnosis*	
Yes	4 (14.3)
No	24 (85.7)
Additional support or benefits received due to the disease*	
Institutionalised care support	4 (30.8)
Financial support	12 (92.3)

* It was possible to give more than one answer

Table 4 presents the WHOQOL domain scores for caregivers of individuals with MLD, compared to the national median for caregivers of people with rare diseases. Across all four domains – Physical health, Psychological health, Social relationships, and Environmental – MLD caregivers reported relatively high average scores. In the Physical health domain, the mean score was 62.3 (SD = 17.8), with 82.1% of caregivers scoring above the national median. The Psychological health domain had a mean of 65.0 (SD = 19.9), with 75% scoring above the median. In the Social relationships domain, the mean was 64.4 (SD = 19.6), with 71.4% exceeding the national median. Finally, the Environmental domain had a mean score of 64.8 (SD = 16.4), with 82.1% of caregivers again scoring above the national benchmark.

**Table 4:** WHOQOL domain scores of MLD caregivers compared to the national median for rare disease caregivers

Domains	Mean (SD)	Number and percentage of caregivers with a score above the national median (*n* = 28)
Physical health	62.3 (17.8)	23 (82.1)
Psychological health	65 (19.9)	21 (75)
Social relationships	64.4 (19.6)	20 (71.4)
Environmental	64.8 (16.4)	23 (82.1)

Table 5 presents Pearson’s correlation coefficients between WHOQOL domains and CFPB-FWBS, including statistical significance and 95% confidence intervals. A strong positive correlation was found between financial well-being and both physical health (*r* = 0.636, *p* < 0.001) and psychological health (*r* = 0.717, *p* < 0.001), indicating that higher financial status is associated with better outcomes in these domains. A moderate positive correlation was also observed with environmental QoL (*r* = 0.567, *p* = 0.002), suggesting a link between financial stability and perceptions of environmental support and living conditions. In contrast, the correlation with social relationships was weaker (*r* = 0.355) and not statistically significant (*p* = ns), suggesting that financial well-being may have a more limited impact on social QoL.

**Table 5:** Pearson’s correlation between WHOQOL domains and CFPB-FWBS

Domains	Pearson’s *r*	*p*	95%CI
Physical health-CFPB Financial Well-Being	0.636	< 0.001	0.345; 0.816
Psychological health-CFPB Financial Well-Being	0.717	< 0.001	0.469; 0.860
Social relationships-CFPB Financial Well-Being	0.355	ns	-0.021; 0.643
Environmental-CFPB Financial Well-Being	0.567	0.002	0.246; 0.776

ns: not significant

Table 6 presents WHOQOL domain scores for mothers and fathers of MLD patients, using a 0–100 scale. Fathers reported higher scores across all domains, with the largest differences in the psychological health (71.9 vs. 59.2) and environmental (71.3 vs. 57.3) domains. The only statistically significant difference was observed in the environmental domain (*p* = 0.05), indicating that fathers perceived a better QoL in this area. Differences in physical health (64.3 vs. 58.6), psychological health (71.9 vs. 59.2), and social relationships (66.5 vs. 62.6) domains were not statistically significant, suggesting largely comparable quality-of-life experiences between mothers and fathers in these areas.

**Table 6:** WHOQOL domain scores by mothers and fathers (transformed scores 0-100)

Domains	Mothers (*n* = 13)	Fathers (*n* = 8)	*P*
Physical health	58.6	64.3	Ns
Psychological health	59.2	71.9	Ns
Social relationships	62.6	66.5	Ns
Environmental	57.3	71.3	0.05

ns: not significant

## Discussion

Our study revealed three important findings regarding the QoL of caregivers of children with MLD in Poland. First, MLD caregivers reported higher-than-average scores in physical health and social relationships domains compared to national medians for caregivers of individuals with RDs [37], and several other rare genetic diseases [40–43]. This is a surprising and counterintuitive result given the heavy caregiving burden and progressive nature of the disease. While several potential explanations may be considered, these should be interpreted with caution as the unexpectedly high QoL scores may also reflect methodological factors discussed below. One possible explanation may be that the caregivers participating in the study were more actively engaged with supportive community networks or advocacy groups, both formal and informal, such as the Borys the Hero Foundation, which may provide social, emotional, or practical resources that buffer stress and promote adaptation [44, 45]. Social connectedness, even when severely limited by caregiving demands, may thus serve as a protective factor and deserves further exploration in rare disease caregiving research.

Another contributing factor may be clinical: MLD leads to a progressive and irreversible loss of motor, cognitive, and communication skills, often requiring complex symptomatic treatment, including polypharmacy and supportive interventions such as gastrostomy or respiratory support. Yet, in our cohort, 69.2% (9 out of 13 patients) received disease-modifying treatment, a notably higher rate than in other studies, e.g., only 29.2% (7 out of 24) in the UK/Ireland [33] and 16.1% (5 out of 31 patients) internationally [32]. Notably, previous studies have shown that pre-symptomatic gene therapy may preserve speech, ambulation, and feeding abilities and reduce caregiving burden [2, 32], whereas the absence of disease-modifying treatment has been associated with greater symptom burden and increased caregiver burden [33]. However, although access to treatment was more common in our cohort than in previous studies, its contribution to the observed QoL outcomes remains uncertain. Given that many patients were already at advanced stages of disease progression, treatment alone is unlikely to fully explain the relatively high QoL scores observed among caregivers.

Additionally, the relatively high QoL scores may also be related to the lower rate of misdiagnoses observed in our cohort: 76.9% had received no prior diagnosis before MLD confirmation. This contrasts with findings by Harrington et al. [13], where 87.5% of patients had experienced at least one misdiagnosis, with caregivers frequently reporting numerous consultations with healthcare professionals before receiving an accurate diagnosis. Meanwhile, studies show that delayed or incorrect diagnosis not only exposes patients to avoidable and costly hospital stays, invasive procedures, and ineffective treatments, often involving frequent medication adjustments that may adversely affect health, but also intensifies the financial and emotional strain experienced by their parents. This suggests that a smoother diagnostic journey may have contributed to an easier adaptation process [42, 43, 46].

Importantly, alternative explanations related to study design should also be considered. Recruitment through a patient advocacy group may have introduced selection bias, potentially favouring more engaged or better-supported families. In addition, response bias cannot be excluded, as caregivers experiencing relatively better coping or QoL may have been more likely to participate. Furthermore, the small sample size limits the generalisability of the findings. Taken together, these methodological factors may provide a more parsimonious explanation for the unexpectedly high QoL scores than the clinical or psychosocial mechanisms discussed above. Therefore, the relatively high QoL scores observed in this study may partly reflect sampling effects rather than true population-level differences and should be interpreted as exploratory findings requiring confirmation in larger cohorts.

At the same time, these findings are consistent with other studies, particularly regarding the observed impact of the diagnostic process on caregiver burden, and align with those of Eichler et al. [34], who reported frequent misdiagnoses among patients with MLD. In the LI and EJ forms, misdiagnoses commonly involve neurological, developmental, and behavioural conditions. Unsurprisingly, Kehrer et al. [30] reported that nearly 75% of MLD families identified “fear of the future” as the most burdensome aspect, and 68% indicated the diagnostic phase and disease progression as the most challenging periods, whereas only one family reported the treatment or terminal stage as most burdensome. Similarly, Koto et al. [47] highlighted persistent geographical and socioeconomic inequalities in access to care, with support often delayed or insufficient, particularly in under-resourced settings.

In our study, 69.2% of caregivers reported that delayed diagnosis had a negative impact on the patient’s health, highlighting the clinical and psychosocial consequences of diagnostic delays. Such delays contribute significantly to caregiver stress and psychological burden, reinforcing the importance of early and accurate diagnosis [27, 32, 47]. Although major aspects of the disease trajectory of MLD have been characterised, diagnostic delays remain common, especially in juvenile-onset cases, where early symptoms often mimic more common developmental or behavioural disorders [13, 17]. Taken together with previous evidence, our findings support the need for improved early detection and more coordinated, multidisciplinary care from the time of diagnosis.

Second, we observed a strong correlation between financial well-being and both physical and psychological QoL, while its effect on social relationships was more limited. This underscores the role of financial stability in maintaining caregivers’ health, especially in the context of chronic caregiving [40, 42, 43]. Disease severity and the child’s dependency level are known predictors of family expenses [48]. Notably, while caregivers exceeded national QoL medians overall, their scores in physical health and psychological health domains were significantly higher than Polish norms [37], suggesting that financial well-being appears to be associated with better caregiver health outcomes, although it does not fully offset the challenges associated with long-term caregiving.

In our cohort, despite relatively favourable financial self-assessment, caregiving remained associated with substantial economic and occupational impact. While rare diseases in general impose a substantial economic burden on caregivers [49–53], MLD-related studies have also demonstrated high healthcare expenditures and significant productivity loss, with over 75% of caregivers reducing or quitting work to meet care demands, and substantial long-term income loss in families of untreated patients, partially mitigated by access to gene therapy [29, 32]. In contrast, in our cohort, 53.6% were employed, and 64.2% rated their financial well-being as medium-high to very high. Still, financial status alone does not fully predict social relationships, as social isolation, loss of leisure activities, and emotional burden may weigh more heavily, especially as MLD progresses [31].

Moreover, the limited correlation between financial status and social QoL points to the complex nature of social burden, which may be influenced more by isolation, stigma, and life disruptions than by material constraints [54, 55]. Caregivers, particularly mothers, face enduring physical, emotional, and financial strain [33, 42, 56]. This is consistent with our findings, where psychological and social domains remained vulnerable despite relatively preserved overall QoL. Previous studies have reported reduced health-related QoL, with high levels of anxiety, depression, sleep disturbance, and chronic fatigue [31, 32].

MLD also exerts sustained pressure on family relationships. More than half of caregivers report marital strain, including emotional distancing, increased conflict, and reduced communication [31, 32]. Thomas et al. [33] report that the relationships with the wider family and friends were often negatively impacted, also influencing siblings’ relationships. Furthermore, siblings often experience grief and loss as their brother or sister irretrievably loses and progresses to suffering.

Third, we identified gender-based disparities in caregiver QoL, with fathers scoring higher than mothers in all domains, especially psychological health and environmental well-being. The difference in environmental QoL was statistically significant, possibly indicating that men experience caregiving differently or receive more external support. These results align with prior studies showing that mothers more often assume primary caregiving roles, face greater emotional and professional disruption [42, 56–60], and have less access to psychological support [31, 61]. Moreover, while participation in leisure and social activities is linked to improved mental health [62], it was reported that mothers who engaged more in such activities reported lower distress and caregiver burden [63]. Fathers may remain more professionally active [56, 64] or more emotionally compartmentalised, leading to better reported well-being, but not necessarily lower burden [42]. Fathers experience emotions similar to those of mothers, but often express them differently: the overlap between their roles as caregivers and providers can make it more difficult for them to process and articulate their feelings. Additionally, men are generally less likely to openly express their emotions [59, 65].

Overall, although the findings should be regarded as exploratory and interpreted with caution given the small sample size and potential sources of bias, they underscore the need for coordinated medical, psychological, and financial support and advocate for early diagnosis, inclusive education, and accessible respite care to better support families living with MLD. Importantly, however, there is a clear lack of data on QoL and caregiver burden in adult patients with MLD. This gap is particularly relevant as many adult patients have families of their own, highlighting the need for further research addressing their specific psychosocial and care needs. Moreover, the burden of MLD in adulthood may involve a substantially different spectrum of disease impact, shaped by age-specific social, familial, and occupational roles and responsibilities [2, 32, 33, 66].

## Limitations

Although to the best of our knowledge, this is the first attempt to assess QoL and caregiver burden in the Polish population of family caregivers of persons with MLD, it has some limitations. Most importantly, the number of study participants was small. However, it should be acknowledged that, given the rarity of the disease and the lack of a rare disease registry in Poland, the exact number of MLD patients and families in the country is unknown. At the same time, the Borys the Hero Foundation, which has helped recruit caregivers, confirmed that the sample covers the majority of known MLD patients in Poland. Thus, the number of 28 responses should be considered high in this context. Nevertheless, the recruitment strategy may have introduced selection bias, as participants were recruited through a patient advocacy group, potentially favouring more engaged or better-supported families. In addition, response bias cannot be excluded, as caregivers experiencing relatively better coping or QoL may have been more likely to participate. Consequently, the findings may not be fully representative of the broader population of MLD caregivers and should be interpreted with caution. Secondly, since the data was gathered through the communication channel of an advocacy group for patients and their families, there is a risk of recruitment bias, as the survey may not have been accessible to parents who are not affiliated with the Foundation, do not use the WhatsApp communication platform, or do not have access to the Internet. Thirdly, although nine males completed the survey, female caregivers still predominated. Fourthly, the self-reported, subjective, and retrospective nature of the survey also limits the study. Fifthly, although the validated GMFC-MLD and ELFC-MLD tools were used, they were completed by caregivers without medical supervision, which carries a risk of error. Sixthly, this study focused on caregivers of children with MLD, and data on QoL and caregiver burden in adult patients with MLD remain limited, highlighting an important area for future research. Seventhly, due to the small sample size, we were unable to perform robust analyses examining the relationships between disease severity, caregiving burden, and caregiver QoL, which represents an important area for future research. Consequently, the findings should be considered exploratory and hypothesis-generating and interpreted with appropriate caution. Finally, to better understand MLD caregivers’ experiences, a more in-depth qualitative study is required.

## Conclusions

In this cohort, MLD was characterised by early-onset, rapidly progressive forms, leading to severe disability and significant caregiver burden. Delayed diagnosis remained common and was frequently reported by caregivers as harming patients’ health. Despite this, many caregivers, especially those with financial support, reported a relatively preserved QoL. However, caregiving demands remained intense, with pronounced impacts on families’ psychological, social, and economic well-being.

Taken together with previous evidence demonstrating the importance of timely diagnosis and early access to disease-modifying therapies in MLD, our findings support the need for improved early detection and coordinated care. Within this broader context, consideration should be given to nationwide (NBS) for MLD, alongside enhanced multidisciplinary care, access to financial and psychological support at diagnosis, investment in caregiver support programmes such as respite care, and the promotion of inclusive education strategies that address the evolving needs of children with MLD. Ensuring a well-coordinated transition of care across life stages is also essential to maintain continuity of support for both patients and caregivers.

## Data Availability

The data supporting this study’s findings are not openly available due to reasons of sensitivity and are available from the corresponding author upon reasonable request. Data are located in controlled access data storage at Poznan University of Medical Sciences.

## Abbreviations

AO: Adult-onset
ARSA: Arylsulfatase A
CFPB-FWBS: The Consumer Financial Protection Bureau’s Financial Well-Being Scale
ELFC-MLD: The expressive language function classification for MLD
EJ: Early juvenile
GMFC-MLD: Gross motor function classification for MLD
LI: Late infantile
LJ: Late juvenile
MLD: Metachromatic leukodystrophy
QoL: Quality of life
WHOQOL-BREF: World Health Organisation Quality of Life-BREF

## References

[1] Gieselmann V, Krägeloh-Mann I. Metachromatic Leukodystrophy – An Update. Neuropediatrics. © Georg Thieme Verlag KG Stuttgart ·. New York. 2010;41:1–6. 10.1055/s-0030-1253412. 20571983

[2] Chang S-C, Eichinger CS, Field P. The natural history and burden of illness of metachromatic leukodystrophy: a systematic literature review. Eur J Med Res. 2024;29:181. 10.1186/s40001-024-01771-1. PMC10946116 38494502

[3] Shaimardanova AA, Chulpanova DS, Solovyeva VV, Mullagulova AI, Kitaeva KV, Allegrucci C, et al. Metachromatic Leukodystrophy: Diagnosis, Modeling, and Treatment Approaches. Front Med (Lausanne). 2020;7:576221. 10.3389/fmed.2020.576221. PMC7606900 33195324

[4] Ługowska A, Wlodarski P, Płoski R, Mierzewska H, Dudzińska M, Matheisel A, et al. Molecular and clinical consequences of novel mutations in the arylsulfatase A gene. Clin Genet. 2009;75:57–64. 10.1111/j.1399-0004.2008.01108.x. 19021637

[5] Trinidad M, Hong X, Froelich S, Daiker J, Sacco J, Nguyen HP, et al. Predicting disease severity in metachromatic leukodystrophy using protein activity and a patient phenotype matrix. Genome Biol. 2023;24:172. 10.1186/s13059-023-03001-z. PMC10360315 37480112

[6] Cesani M, Lorioli L, Grossi S, Amico G, Fumagalli F, Spiga I, et al. Mutation Update of ARSA and PSAP Genes Causing Metachromatic Leukodystrophy. Hum Mutat. 2016;37:16–27. 10.1002/humu.22919. 26462614

[7] Kolnikova M, Jungova P, Skopkova M, Foltan T, Gasperikova D, Mattosova S, et al. Late Infantile Metachromatic Leukodystrophy Due to Novel Pathogenic Variants in the PSAP Gene. J Mol Neurosci. 2019;67:559–63. 10.1007/s12031-019-1259-7. 30632081

[8] Kehrer C, Elgün S, Raabe C, Böhringer J, Beck-Wödl S, Bevot A, et al. Association of Age at Onset and First Symptoms With Disease Progression in Patients With Metachromatic Leukodystrophy. Neurology. 2021;96:e255–66. 10.1212/WNL.0000000000011047. 33046606

[9] Ługowska A, Ponińska J, Krajewski P, Broda G, Płoski R. Population carrier rates of pathogenic ARSA gene mutations: is metachromatic leukodystrophy underdiagnosed? PLoS ONE. 2011;6:e20218. 10.1371/journal.pone.0020218. PMC3112151 21695197

[10] Chang S-C, Bergamasco A, Bonnin M, Bisonó TA, Moride Y. A systematic review on the birth prevalence of metachromatic leukodystrophy. Orphanet J Rare Dis. 2024;19:80. 10.1186/s13023-024-03044-w. PMC10880320 38383398

[11] Gieselmann V. Metachromatic leukodystrophy: genetics, pathogenesis and therapeutic options. Acta Paediatr. 2008;97:15–21. 10.1111/j.1651-2227.2008.00648.x. 18339182

[12] Chauhan NS, Sharma M, Bhardwaj A. Classical case of late-infantile form of metachromatic leukodystrophy. J Neurosci Rural Pract. 2016;7:473–5. 10.4103/0976-3147.181482. PMC4898128 27365977

[13] Harrington M, Whalley D, Twiss J, Rushton R, Martin S, Huynh L, et al. Insights into the natural history of metachromatic leukodystrophy from interviews with caregivers. Orphanet J Rare Dis. 2019;14:89. 10.1186/s13023-019-1060-2. PMC6489348 31036045

[14] Kehrer C, Blumenstock G, Raabe C, Krägeloh-Mann I. Development and reliability of a classification system for gross motor function in children with metachromatic leucodystrophy. Dev Med Child Neurol. 2011;53:156–60. 10.1111/j.1469-8749.2010.03821.x. 21087233

[15] Martin S, Harris N, Romanus D. Evaluating meaningful changes in physical functioning and cognitive declines in metachromatic leukodystrophy: a caregiver interview study. J Patient Rep Outcomes. 2023;7:70. 10.1186/s41687-023-00595-7. PMC10352216 37458805

[16] Banks JL, Marotta CA. Outcomes validity and reliability of the modified Rankin scale: implications for stroke clinical trials: a literature review and synthesis. Stroke. 2007;38:1091–6. 10.1161/01.STR.0000258355.23810.c6. 17272767

[17] Kehrer C, Groeschel S, Kustermann-Kuhn B, Bürger F, Köhler W, Kohlschütter A, et al. Language and cognition in children with metachromatic leukodystrophy: onset and natural course in a nationwide cohort. Orphanet J Rare Dis. 2014;9:18. 10.1186/1750-1172-9-18. PMC3922034 24499656

[18] Kehrer C, Blumenstock G, Gieselmann V, Krägeloh-Mann I, GERMAN LEUKONET. The natural course of gross motor deterioration in metachromatic leukodystrophy. Dev Med Child Neurol. 2011;53:850–5. 10.1111/j.1469-8749.2011.04028.x. 21707604

[19] Sevin C, Mochel F. Hematopoietic stem cell transplantation in leukodystrophies. Handb Clin Neurol. 2024;204:355–66. 10.1016/B978-0-323-99209-1.00017-X. 39322389

[20] Fumagalli F, Calbi V, Sora MGN, Sessa M, Baldoli C, Rancoita PMV, et al. Lentiviral haematopoietic stem-cell gene therapy for early-onset metachromatic leukodystrophy: long-term results from a non-randomised, open-label, phase 1/2 trial and expanded access. Lancet Elsevier. 2022;399:372–83. 10.1016/S0140-6736(21)02017-1. PMC8795071 35065785

[21] Messina M, Gissen P. Atidarsagene autotemcel for metachromatic leukodystrophy. Drugs Today (Barc). 2023;59:63–70. 10.1358/dot.2023.59.2.3461911. 36811406

[22] Horgan C, Watts K, Ram D, Rust S, Hutton R, Jones S, et al. A retrospective cohort study of Libmeldy (atidarsagene autotemcel) for MLD: What we have accomplished and what opportunities lie ahead. JIMD Rep. 2023;64:346–52. 10.1002/jmd2.12378. PMC10494509 37701322

[23] van Rappard DF, Boelens JJ, Wolf NI. Metachromatic leukodystrophy: Disease spectrum and approaches for treatment. Best Pract Res Clin Endocrinol Metab. 2015;29:261–73. 10.1016/j.beem.2014.10.001. 25987178

[24] Hong X, Daiker J, Sadilek M, Ruiz-Schultz N, Kumar AB, Norcross S, et al. Toward newborn screening of metachromatic leukodystrophy: results from analysis of over 27,000 newborn dried blood spots. Genet Med. 2021;23:555–61. 10.1038/s41436-020-01017-5. PMC10395749 33214709

[25] Wu THY, Brown HA, Church HJ, Kershaw CJ, Hutton R, Egerton C, et al. Improving newborn screening test performance for metachromatic leukodystrophy: Recommendation from a pre-pilot study that identified a late-infantile case for treatment. Mol Genet Metab. 2024;142:108349. 10.1016/j.ymgme.2024.108349. 38458124

[26] Laugwitz L, Schoenmakers DH, Adang LA, Beck-Woedl S, Bergner C, Bernard G, et al. Newborn screening in metachromatic leukodystrophy - European consensus-based recommendations on clinical management. Eur J Paediatr Neurol. 2024;49:141–54. 10.1016/j.ejpn.2024.03.003. 38554683

[27] Morton G, Thomas S, Roberts P, Clark V, Imrie J, Morrison A. The importance of early diagnosis and views on newborn screening in metachromatic leukodystrophy: results of a Caregiver Survey in the UK and Republic of Ireland. Orphanet J Rare Dis. 2022;17:403. 10.1186/s13023-022-02550-z. PMC9635117 36329444

[28] Shih ST, Farrar MA, Wiley V, Chambers G. Newborn screening for spinal muscular atrophy with disease-modifying therapies: a cost-effectiveness analysis. J Neurol Neurosurg Psychiatry. 2021;92:1296–304. 10.1136/jnnp-2021-326344. 34321343

[29] Bean K, Olaye A, Velikanova R, Miller B, Howle K, Wilds A, et al. EE45 The societal costs of Metachromatic Leukodystrophy (MLD) in the Netherlands. Value in Health. Elsevier; 2022;25:S344. 10.1016/j.jval.2022.04.299.

[30] Kehrer C, Bevot A, Martin P, Raabe C, Gregor S, Krägeloh-Mann I, et al. Healthcare utilization and disease burden in children with metachromatic leukodystrophy in Germany. Orphanet J Rare Dis. 2025;20:242. 10.1186/s13023-025-03637-z. PMC12100800 40410895

[31] Ammann-Schnell L, Groeschel S, Kehrer C, Frölich S, Krägeloh-Mann I. The impact of severe rare chronic neurological disease in childhood on the quality of life of families-a study on MLD and PCH2. Orphanet J Rare Dis. 2021;16:211. 10.1186/s13023-021-01828-y. PMC8111977 33971942

[32] Sevin C, Barth M, Wilds A, Afriyie A, Walz M, Dillon A, et al. An international study of caregiver-reported burden and quality of life in metachromatic leukodystrophy. Orphanet J Rare Dis. 2022;17:329. 10.1186/s13023-022-02501-8. PMC9438185 36056437

[33] Thomas S, Morrison A, Morton G, Roberts P, Clark V, Imrie J. The burden of disease in metachromatic leukodystrophy: results of a caregiver survey in the UK and Republic of Ireland. Orphanet J Rare Dis. 2024;19:87. 10.1186/s13023-023-03001-z. PMC10895743 38403596

[34] Nowak JK, Walkowiak J. Study designs in medical research and their key characteristics. J Med Sci. 2023;92:e928–928. 10.20883/medical.e928.

[35] Frisch N, Atherton P, Doyle-Waters MM, MacLeod MLP, Mallidou A, Sheane V, et al. Patient-oriented research competencies in health (PORCH) for researchers, patients, healthcare providers, and decision-makers: results of a scoping review. Res Involv Engagem. 2020;6:4. 10.1186/s40900-020-0180-0. PMC7011284 32055415

[36] Sawicka-Gutaj N, Gruszczyński D, Guzik P, Mostowska A, Walkowiak J. Publication ethics of human studies in the light of the Declaration of Helsinki – a mini-review. JMS. 2022;91:e700–700. 10.20883/medical.e700.

[37] Jabkowski P, Domaradzki J, Walkowiak D. Quality of Life Among Family Caregivers of Individuals With Rare Diseases: Web-Based Population Study on the Validity and Reliability of the Polish World Health Organization Quality of Life-BREF Questionnaire. JMIR Public Health Surveill. 2025;11:e72590. 10.2196/72590. PMC12294646 40680179

[38] Measuring financial well-being. A guide to using the CFPB financial well-being scale [Internet]. Consumer Financial Protection Bureau. 2024 [cited 2024 Sept 2]. https://www.consumerfinance.gov/data-research/research-reports/financial-well-being-scale/. Accessed 2 Sept 2024.

[39] Walkowiak D, Jabkowski P, Domaradzki J. Validation and psychometric properties of the Consumer Financial Protection Bureau Financial Well-Being Scale (CFPB-FWBS) for Polish family caregivers of individuals with rare diseases. J Med Sci. 2025;94:e1428–1428. 10.20883/medical.e1428.

[40] Domaradzki J, Walkowiak D. Quality of life and caregiving burden associated with parenting a person with Duchenne/Becker muscular dystrophy in Poland. Orphanet J Rare Dis. 2024;19:450. 10.1186/s13023-024-03481-7. PMC11608484 39616352

[41] Walkowiak D, Domaradzki J. The impact of 22q11.2 deletion syndrome on caregivers: assessing quality of life and burden. Orphanet J Rare Dis. 2025;20:264. 10.1186/s13023-025-03790-5. PMC12126857 40452047

[42] Walkowiak D, Domaradzki J. Caregiving Burden and Quality of Life Among Parents of Individuals With Angelman Syndrome: Gender Differences and the Impact of Financial Well-Being. Pediatr Neurol. 2025;169:31–9. 10.1016/j.pediatrneurol.2025.05.005. 40449417

[43] Walkowiak D, Domaradzki J. Perception of psychosocial burden in mothers of children with rare pediatric neurological diseases. Sci Rep. 2025;15:6295. 10.1038/s41598-025-87251-w. PMC11845487 39984547

[44] Ashtari S, Taylor A. Patients With Rare Diseases and the Power of Online Support Groups: Implications for the Medical Community. JMIR Form Res. 2023;7:e41610. 10.2196/41610. PMC10540027 37707878

[45] Delisle VC, Gumuchian ST, Rice DB, Levis AW, Kloda LA, Körner A, et al. Perceived Benefits and Factors that Influence the Ability to Establish and Maintain Patient Support Groups in Rare Diseases: A Scoping Review. Patient. 2017;10:283–93. 10.1007/s40271-016-0213-9. 28004275

[46] Domaradzki J, Walkowiak D. Ultra-rare ultra-care: Assessing the impact of caring for children with ultra rare diseases. Eur J Paediatr Neurol. 2024;48:78–84. 10.1016/j.ejpn.2023.12.003. 38071849

[47] Mohajer A, Sevagamoorthy A, Bean K, Mutua S, Pang F, Adang LA. Characterizing Diagnostic Delays in Metachromatic Leukodystrophy: A Real-World Data Approach. J Inherit Metab Dis. 2025;48:e70049. 10.1002/jimd.70049. PMC12130617 40457575

[48] Buckle N, Doyle O, Kodate N, Kinch M, Somanadhan S. Caregiver-Reported Economic Impacts of Pediatric Rare Diseases-A Scoping Review. Healthc (Basel). 2024;12:2578. 10.3390/healthcare12242578. PMC11727781 39766005

[49] Andreu P, Atay NJ, Piccinini E, Chiesi G, Cioffi G. Rare disease burden of care and the economic impact on citizens in Germany, France and Italy.

[50] Angelis A, Tordrup D, Kanavos P. Socio-economic burden of rare diseases: A systematic review of cost of illness evidence. Health Policy. 2015;119:964–79. 10.1016/j.healthpol.2014.12.016. 25661982

[51] Chung CCY, Ng NYT, Ng YNC, Lui ACY, Fung JLF, Chan MCY, et al. Socio-economic costs of rare diseases and the risk of financial hardship: a cross-sectional study. Lancet Reg Health West Pac. 2023;34:100711. 10.1016/j.lanwpc.2023.100711. PMC10240356 37283971

[52] Cavazza M, Kodra Y, Armeni P, De Santis M, López-Bastida J, Linertová R, et al. Social/economic costs and health-related quality of life in patients with Duchenne muscular dystrophy in Europe. Eur J Health Econ Springer Berlin Heidelberg. 2016;17:19–29. 10.1007/s10198-016-0782-5. 27038625

[53] Walkowiak D, Pospieszyńska-Martysiuk K, Dianow H, Węgrzyn J, Domaradzki J. Measuring economic burden in families of individuals with Angelman Syndrome in Poland: a caregivers’ survey. Orphanet J Rare Dis. 2025;21:24. 10.1186/s13023-025-04183-4. PMC12829253 41469742

[54] Adama EA, Arabiat D, Foster MJ, Afrifa-Yamoah E, Runions K, Vithiatharan R, et al. The psychosocial impact of rare diseases among children and adolescents attending mainstream schools in Western Australia. Int J Incl Educ Routledge. 2023;27:1273–86. 10.1080/13603116.2021.1888323.

[55] Baynam G, Gomez R, Jain R. Stigma associated with genetic testing for rare diseases-causes and recommendations. Front Genet. 2024;15:1335768. 10.3389/fgene.2024.1335768. PMC11024281 38638122

[56] Walkowiak D, Domaradzki J, Mozrzymas R, Korycińska-Chaaban D, Duś-Żuchowska M, Didycz B, et al. Professional activity, gender and disease-related emotions: The impact on parents’ experiences in caring for children with phenylketonuria. Mol Genet Metab Rep. 2023;36:100992. 10.1016/j.ymgmr.2023.100992. PMC10368909 37502286

[57] Chiarotti F, Kodra Y, De Santis M, Bellenghi M, Taruscio D, Carè A, et al. Gender and burden differences in family caregivers of patients affected by ten rare diseases. Ann Ist Super Sanita. 2023;59:122–31. 10.4415/ANN_23_02_05. 37337987

[58] Chu S-Y, Wen C-C, Weng C-Y. Gender Differences in Caring for Children with Genetic or Rare Diseases: A Mixed-Methods Study. Child (Basel). 2022;9:627. 10.3390/children9050627. PMC9139271 35626804

[59] Cardinali P, Migliorini L, Rania N. The Caregiving Experiences of Fathers and Mothers of Children With Rare Diseases in Italy: Challenges and Social Support Perceptions. Front Psychol. 2019;10:1780. 10.3389/fpsyg.2019.01780. PMC6690318 31428029

[60] Pelchat D, Lefebvre H, Levert M-J. Gender differences and similarities in the experience of parenting a child with a health problem: current state of knowledge. J Child Health Care. 2007;11:112–31. 10.1177/1367493507076064. 17494986

[61] Boettcher J, Boettcher M, Wiegand-Grefe S, Zapf H. Being the Pillar for Children with Rare Diseases-A Systematic Review on Parental Quality of Life. Int J Environ Res Public Health. 2021;18:4993. 10.3390/ijerph18094993. PMC8125857 34066738

[62] Chen S, Wang Y, Zhu L, Feng Z, Gong S, Dong D. Social activity as a mediator between social support and psychological quality of life among people with rare diseases: A national repetitive cross-sectional study. J Psychiatr Res. 2022;150:147–52. 10.1016/j.jpsychires.2022.03.042. 35378487

[63] Cremers CH, Fischer MJ, Kruitwagen-van Reenen ET, Wadman RI, Vervoordeldonk JJ, Verhoef M, et al. Participation and mental well-being of mothers of home-living patients with spinal muscular atrophy. Neuromuscul Disord. 2019;29:321–9. 10.1016/j.nmd.2019.02.011. 30954388

[64] von der Lippe C, Neteland I, Feragen KB. Children with a rare congenital genetic disorder: a systematic review of parent experiences. Orphanet J Rare Dis. 2022;17:375. 10.1186/s13023-022-02525-0. PMC9575260 36253830

[65] de Lucca SA, Petean EBL. Fatherhood: experiences of fathers of boys diagnosed with Duchenne Muscular Dystrophy. Cien Saude Colet. 2016;21:3081–9. 10.1590/1413-812320152110.20302016. 27783781

[66] Mandia D, Dufour J, Fenu S, Salsano E, Benzoni C, Sellal F, et al. Natural history of adult-onset metachromatic leukodystrophy. J Neurol. 2025;272:303. 10.1007/s00415-025-12997-2. PMC11961487 40167764

